# Unique Effects of (R)-Ketamine Compared to (S)-Ketamine on EEG Theta Power in Rats

**DOI:** 10.3390/ph17020194

**Published:** 2024-02-01

**Authors:** Dóra Pothorszki, Szabolcs Koncz, Dóra Török, Noémi Papp, György Bagdy

**Affiliations:** 1 Department of Pharmacodynamics, Faculty of Pharmaceutical Sciences, Semmelweis University, 1089 Budapest, Hungary; pothorszki.dora@phd.semmelweis.hu (D.P.); koncz.szabolcs@semmelweis.hu (S.K.); torok.dora@phd.semmelweis.hu (D.T.); papp.noemi@pharma.semmelweis-univ.hu (N.P.); 2 NAP3.0-SE Neuropsychopharmacology Research Group, Hungarian Brain Research Program, Semmelweis University, 1089 Budapest, Hungary

**Keywords:** electroencephalography, theta rhythm, arketamine, esketamine, REM sleep, quantitative EEG, cognition, memory, neuroplasticity, antidepressant

## Abstract

Differences in the pharmacological effects of (S)-ketamine and (R)-ketamine are at the focus of research. Clinical data and our rat studies confirmed the antidepressant effect of (S)- but not (R)-ketamine, with similar differences in quantitative electroencephalogram (EEG) and sleep effects. In contrast, studies mainly on mice showed some stronger, preferable effects of (R)-ketamine. EEG theta (5–9 Hz) rhythm originates from the hippocampus, and its power is associated with cognitive functions, attention, and decreased anxiety. To find a brain parameter that is not associated with the antidepressant effect of drugs and may confirm potent in vivo effects of (R)-ketamine in rats, theta EEG power-inducing effects of the two enantiomers were measured and compared for 23 h. EEG-equipped Wistar rats were treated with (R)-ketamine (7.5, 15, 30 mg/kg i.p.), (S)-ketamine (7.5 and 15 mg/kg i.p.), or vehicle at the beginning of the passive phase. Frontoparietal EEG, electromyogram, and motor activity were recorded. (R)-ketamine but not (S)-ketamine dose-dependently increased EEG theta power during wakefulness and rapid eye movement (REM) sleep for 23 h. These results suggest that (R)-ketamine has an effect on a hippocampal function that was not affected by (S)-ketamine and may be associated with neural plasticity and memory encoding.

## 1. Introduction

(R,S)-ketamine is an N-methyl-d-aspartate (NMDA) receptor antagonist consisting of two enantiomers, (R)-ketamine and (S)-ketamine. The two enantiomers have distinct pharmacological profiles. (S)-ketamine has a 4-fold higher binding affinity for NMDA receptors than (R)-ketamine [1], and it has been suggested that they affect serotonergic and dopaminergic neurotransmission in the prefrontal cortex (PFC) differently [2]. Furthermore, (S)-ketamine has been shown to bind to the mu-opioid receptor (MOR) (Ki: 7 ± 3 μM) and kappa-opioid receptor (KOR) (Ki: 14 ± 7 μM) with higher affinity than (R)-ketamine (Ki: 19 ± 5 μM, 40 ± 10 μM, respectively), while (R)-ketamine showed greater affinity for sigma-1 and sigma-2 receptors (Ki  =  27  ±  3 µM, Ki  =  0.5  ±  0.1 mM, respectively) than (S)-ketamine (131  ±  15 µM, 2.8  ±  0.7 mM, respectively) [3]. (S)-ketamine has recently been approved for treatment-resistant depression by the Food and Drug Administration (FDA) and the European Medicines Agency (EMA), while (R)-ketamine had no significant antidepressant effect compared to placebo in recent clinical studies, suggesting caution when interpreting its therapeutic potential [4]. In animal models, the racemic mixture and both enantiomers have been shown to cause rapid-onset antidepressant effects, with the addition of (R)-ketamine having a more favorable side-effect profile than (S)-ketamine since it does not cause psychotomimetic effects [5]. Recently, we have shown that 15 mg/kg i.p. (S)-ketamine but not (R)-ketamine has acute effects on depression-like behavior and sleep–wake architecture, which is in line with the clinical data [6]. To elaborate, (S)-ketamine decreased REM sleep time at two and three hours following administration, while it decreased NREM sleep and increased wakefulness in the first two hours. Moreover, it increased EEG delta (1–4 Hz) power during NREM sleep. (R)-ketamine had no effects on sleep–wake architecture nor on EEG delta power. In the behavioral experiment, chronically stressed rats were exposed to the forced swimming test. The results showed that (S)-ketamine but not (R)-ketamine significantly decreased the immobility time, suggesting that only (S)-ketamine has antidepressant-like effects at this dose.

Beyond its antidepressant effect, however, other potential therapeutic applications of (R)-ketamine have been proposed. Namely, (R)-ketamine but not (S)-ketamine could improve cognitive deficit in mice caused by phenyl cyclohexyl piperidine (PCP), suggesting that (R)-ketamine could be a potential therapeutic drug for cognitive impairments [7]. Moreover, (R)-ketamine but not (S)-ketamine could attenuate 1-methyl-4-phenyl-1,2,3,6-tetrahydropyridine (MPTP)-induced reduction of dopamine transporter (DAT) and tyrosine hydroxylase (TH) in the striatum of mice, suggesting that (R)-ketamine may be a new prophylactic or therapeutic drug for Parkinson’s disease [8]. Moreover, repeated administration of (R)-ketamine could improve the measured parameters in multiple sclerosis (MS) animal models [9,10].

Theta rhythm is generated mostly by the hippocampus region of the mammalian brain [11]. Early studies revealed that theta rhythm has an important role in episodic memory and visuospatial processing. According to more recent findings, theta is involved in a wide range of cognitive-affective functions, for example, in sensory and motor processing, passive and active attention, and navigation [12]. Theta activity is a prominent feature of rapid eye movement (REM) sleep in humans and rodents; however, it is likely that their generation and regulation in REM sleep and wakefulness might serve distinct, though possibly related functions [13]. Theta power in rats, measured by cortical surface electrodes, shows a hippocampal function not affected by chronic antidepressant, e.g., fluoxetine or escitalopram, treatments [14,15].

Our aim in this study was to find a brain parameter that is not associated with depression and the antidepressant effect of drugs and may confirm potent in vivo effects of (R)-ketamine in rats. Therefore, theta EEG power-inducing effects of different doses of the two enantiomers were compared for 23 h.

## 2. Results

### 2.1. Effects of (R)-ketamine and (S)-ketamine on qEEG during Wakefulness

**Figure 1:**
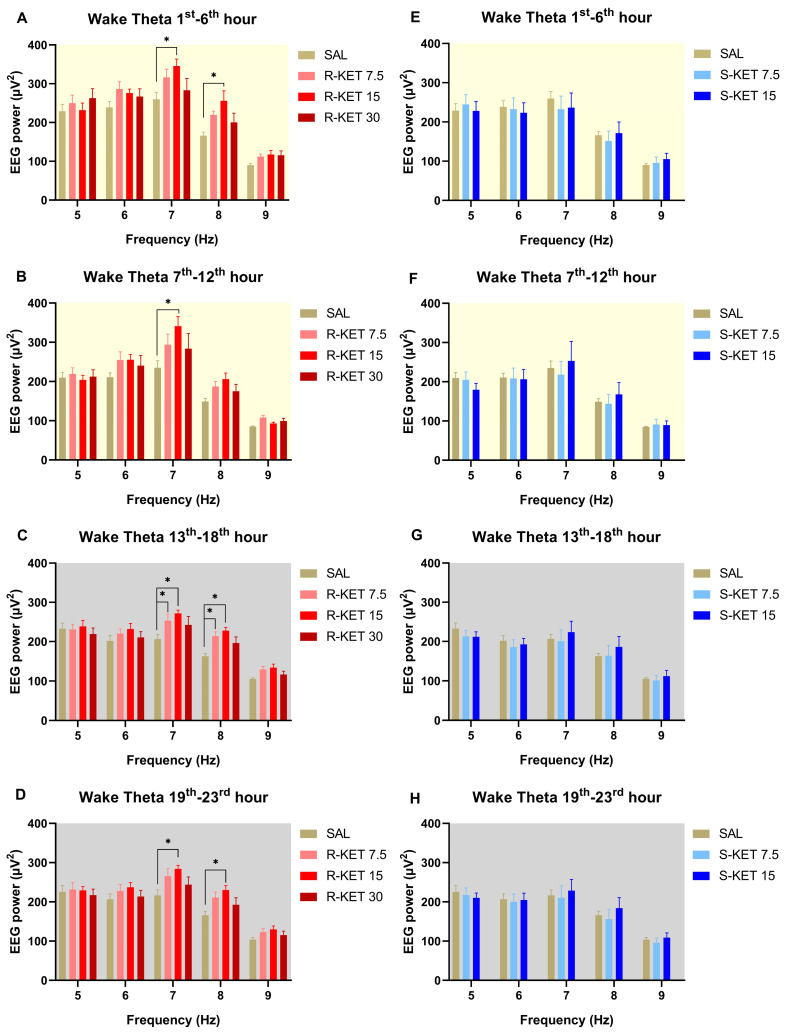
Effects of (R)-ketamine [7.5, 15, 30 mg/kg i.p., (R)-KET], (S)-ketamine [7.5, 15 mg/kg i.p., (S)-KET], and vehicle (saline, SAL) on EEG theta power (5–9 Hz) for 23 h following administration shown in 6-h periods during wakefulness. Effects of (R)-ketamine on theta power are shown in the (**A**) 1st–6th hours, (**B**) 7th–12th hours, (**C**) 13th–18th hours, and (**D**) 19th–23rd hours after administration. Effects of (S)-ketamine on theta power are shown in the (**E**) 1st–6th hours, (**F**) 7th–12th hours, (**G**) 13th–18th hours, and (**H**) 19th–23rd hours after administration. Significant results are indicated by * (compared with vehicle, *p* < 0.05). The background colors indicate the passive (yellow—lights on) and the active (grey—lights off) phases. Results are presented as mean ± SEM (n = 6–8 rats per group).

(R)-ketamine showed a dose-dependent and sustained theta power increasing effect during wakefulness for 23 h after administration (two-way ANOVA treatment effect: 1st–6th: F (3, 120) = 7.354 *p* = 0.0001 Figure 1A, 7th–12th: F (3, 120) = 5.727 *p* = 0.0011 Figure 1B, 13th–18th: F (3, 120) = 8.982 *p* < 0.0001 Figure 1C and 19th–23rd: F (3, 120) = 7.345 *p* = 0.0001 Figure 1D). The most prominent effects were seen at the frequency band of 7 and 8 Hz. Namely, Bonferroni post hoc comparisons showed that 15 mg/kg i.p. (R)-ketamine significantly elevated theta power in the 1st–6th, 7th–12th, 13th–18th, and 19th–23rd hour periods following administration (Figure 1A–D), while 7.5 mg/kg i.p. (R)-ketamine significantly increased theta power in the 13th–18th hour period after receiving the treatment (Figure 1C). The highest, 30 mg/kg i.p. dose, showed a trend in theta power elevation; however, this effect did not reach statistical significance in the Bonferroni post hoc comparison.

**Figure 2:**
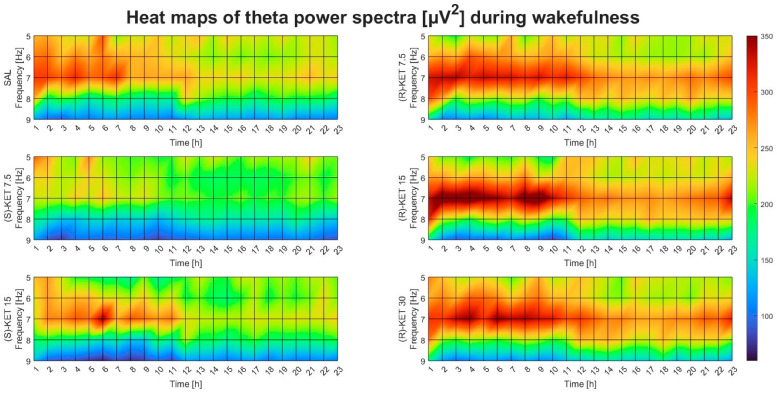
Effects of (R)-ketamine [7.5, 15, 30 mg/kg i.p., (R)-KET], (S)-ketamine [7.5, 15 mg/kg i.p., (S)-KET], and vehicle (saline, SAL) on EEG theta power (5–9 Hz) for 23 h following administration during wakefulness.

Theta power was not altered by either (S)-ketamine doses (7.5 or 15 mg/kg i.p.) in the first 23 h after administration during wakefulness (two-way ANOVA treatment effect: 1st–6th: F (2, 95) = 0.07693 *p* = 0.9260 Figure 1E, 7th–12th: F (2, 95) = 0.0933 *p* = 0.9110 Figure 1F, 13th–18th: F (2, 95) = 0.6346 *p* = 0.5324 Figure 1G and 19th–23rd: F (2, 95) = 0.4685 *p* = 0.6274 Figure 1H). Hourly breakdowns of the effects of (R)-ketamine and (S)-ketamine on theta power during wakefulness for the whole 23-h period can be seen in Figure 2.

### 2.2. Effects of (R)-ketamine and (S)-ketamine on qEEG during REM Sleep

**Figure 3:**
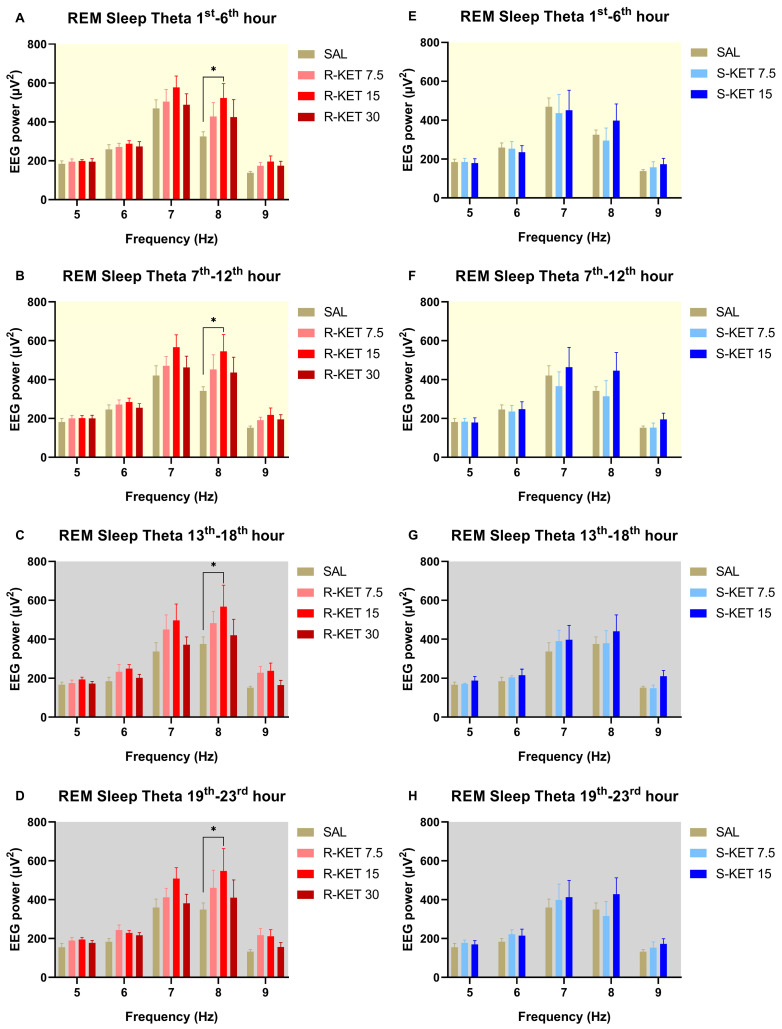
Effects of (R)-ketamine [7.5, 15, 30 mg/kg i.p., (R)-KET], (S)-ketamine [7.5, 15 mg/kg i.p., (S)-KET], and vehicle (saline, SAL) on EEG theta power (5–9 Hz) for 23 h following administration shown in 6-h periods during REM sleep. Effects of (R)-ketamine on theta power are shown in the (**A**) 1st–6th hours, (**B**) 7th–12th hours, (**C**) 13th–18th hours, and (**D**) 19th–23rd hours after administration. Effects of (S)-ketamine on theta power are shown in the (**E**) 1st–6th hours, (**F**) 7th–12th hours, (**G**) 13th–18th hours, and (**H**) 19th–23rd hours after administration. Significant results are indicated by * (compared with vehicle, *p* < 0.05). The background colors indicate the passive (yellow—lights on) and the active (grey—lights off) phases. Results are presented as mean ± SEM (n = 6–8 rats per group).

(R)-ketamine dose-dependently increased theta power during REM sleep for 23 h after administration (two-way ANOVA treatment effect: 1st–6th: F (3, 120) = 3.207 *p* = 0.0256 Figure 3A, 7th–12th: F (3, 120) = 4.283 *p* = 0.0066 Figure 3B, 13th–18th: F (3, 120) = 5.357 *p* = 0.0017 Figure 3C and 19th–23rd: F (3, 120) = 4.725 *p* = 0.0038 Figure 3D). The most prominent effects were seen at the frequency band of 8 Hz. Namely, Bonferroni post hoc comparisons showed that 15 mg/kg i.p. (R)-ketamine significantly elevated theta power in the 1st–6th, 7th–12th, 13th–18th, and 19th–23rd hour periods following administration (Figure 3A–D).

**Figure 4:**
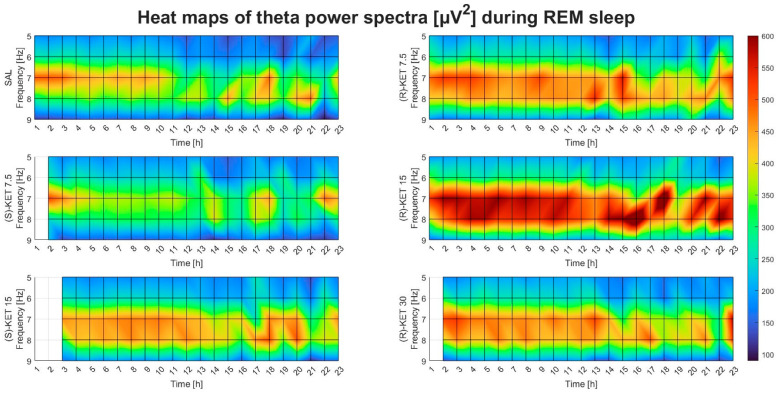
Effects of (R)-ketamine [7.5, 15, 30 mg/kg i.p., (R)-KET], (S)-ketamine [7.5, 15 mg/kg i.p., (S)-KET], and vehicle (saline, SAL) on EEG theta power (5–9 Hz) for 23 h following administration during REM sleep. White boxes indicate that the given treatment caused REM sleep suppression; to such a degree that quantitative EEG analysis could not be performed.

Neither (S)-ketamine doses (7.5 or 15 mg/kg i.p.) altered theta power in the first 23 h after administration during REM sleep (two-way ANOVA treatment effect: 1st–6th: F (2, 95) = 0.2514 *p* = 0.7783 Figure 3E, 7th–12th: F (2, 95) = 1.542 *p* = 0.2192 Figure 3F, 13th–18th: F (2, 95) = 1.773 *p* = 0.1753 Figure 3G and 19th–23rd: F (2, 90) = 1.207 *p* = 0.3040 Figure 3H). The effects of (R)-ketamine and (S)-ketamine on theta power during REM sleep for the whole 23-h period in hourly breakdowns can be seen in Figure 4.

## 3. Discussion

In this study, we have shown that (R)-ketamine but not (S)-ketamine dose-dependently and sustainably (up to 23 h following administration) increased qEEG theta power during wakefulness and REM sleep. This effect was phase-independent; it appeared both in the passive (light) and in the active (dark) phase of the animals.

The hippocampus, where cortical theta oscillations are primarily generated, represents a neural substrate of neural plasticity, the nervous system’s capacity to adapt to both internal and external stimuli by rearranging its connections and structure [11]. An earlier animal study found that (R)-ketamine and (S)-ketamine could upregulate synaptogenesis in the PFC and in the CA3 and dentate gyrus (DG) of the hippocampus by increasing the probability of glutamate release after administration, which increases the α-amino-3-hydroxy-5-methyl-4-isoxazolepropionic acid receptor (AMPA) receptor throughput. Through this effect, brain-derived neurotrophic factor (BDNF) release is increased, which binds to TrkB (tropomyosin receptor kinase B) receptors on the postsynaptic neuron. The difference is that in the case of (S)-ketamine, BDNF release is facilitated through preferential binding to NMDA receptors expressed in GABAergic interneurons, which leads to the depolarization of cortical excitatory neurons, while (R)-ketamine appears to facilitate immune modulation by affecting microglial signaling. Moreover, downstream of the BDNF–TrkB signaling, (R)-ketamine and (S)-ketamine have been proposed to exert their synaptogenesis upregulating effect on different pathways. Namely, (R)-ketamine exerts its effect via the activation of the extracellular signal-regulated kinase (ERK) pathway, while (S)-ketamine acts through the disinhibition of mechanistic target of rapamycin (mTORC1) signaling [5,16]. In our previous animal study, we found that (S)-ketamine but not (R)-ketamine showed antidepressant-like effects in sleep architecture and EEG delta power [6], while in this study, we found that only (R)-ketamine increased EEG theta power significantly. Our results, therefore, might support the theory that the two enantiomers exert their pharmacological effects through different pathways, and (R)-ketamine’s potential therapeutic effect may be connected to the increased theta activity.

Preclinical evidence showed that (R)-ketamine but not (S)-ketamine could improve PCP-induced cognitive deficit in mice. This beneficial effect of (R)-ketamine could be blocked by ANA-12 pretreatment, a TrkB inhibitor, which suggests that this effect is possibly exerted via the activation of the BDNF-TrkB signaling, as well. Based on this, (R)-ketamine has been suggested as a potential therapeutic drug for disorders with cognitive impairment [7,17]. Theta rhythm has been shown to have a crucial role in cognitive processes (learning and memory) and in the control of complex behaviors [11]. Therefore, our results on (R)-ketamine’s theta-enhancing effect shown in this study may support further investigations regarding its potential use in disorders with cognitive impairment.

A study with an animal model of Parkinson’s disease showed that subsequent repeated intranasal administration of both enantiomers attenuated MPTP-induced reduction of DAT, although (R)-ketamine’s effect was more potent than that of (S)-ketamine [8]. In a frequently used animal model for MS, called the experimental autoimmune encephalomyelitis (EAE), (R)-ketamine could improve EAE scores and pathological alterations in the spinal cord of mice [9]. In another animal model used for MS, cuprizone (CPZ)-treated mice’s demyelination was decreased by (R)-ketamine [10]. An earlier study revealed that in a working memory-related task, MS patients’ right hippocampal maximum theta power increase was attenuated compared to healthy subjects [18]. Our results on (R)-ketamine’s theta-enhancing effect, therefore, support further investigations regarding the drug’s potential effect on MS. In both MPTP and CPZ animal models, pretreatment with TrkB antagonist ANA-12 significantly blocked the effect of (R)-ketamine, suggesting the role of BDNF-TrkB signaling activation in its mechanism of action [8,10].

Pharmacokinetic experiments of (R)-ketamine and (S)-ketamine showed that the exposure levels of both compounds in the brain and CSF were approximately the same after administration. Moreover, 24 h following administration, neither of the compounds were present in the brain or CSF, suggesting that the differences between (R)-ketamine and (S)-ketamine cannot be ascribed to differences in their pharmacokinetic profiles [19].

Differences in the effects on theta power between the two enantiomers could be explained by their different pharmacological profiles. It is well-known that (S)-ketamine has a 2–4 times higher binding affinity for NMDA receptors [1]. The administered (R)-ketamine doses showed a bell-shaped response curve: the most potent effects were seen at 15 mg/kg i.p. (R)-ketamine. Neither (S)-ketamine (7.5 mg/kg, 15 mg/kg) nor a high dose of (R)-ketamine (30 mg/kg) caused a significant increase in theta power despite their stronger NMDA receptor antagonism. Indeed, NMDAR antagonists reduce hippocampal theta power in freely moving rats [20]. This suggests that (R)-ketamine’s theta power-increasing effect is not caused by NMDA receptor antagonism; it is mediated by other receptors. For example, an earlier study showed that (R)-ketamine had a greater affinity for sigma-1 and sigma-2 receptors (Ki  =  27  ±  3 µM, Ki  =  0.5  ±  0.1 mM, respectively) than (S)-ketamine (131  ±  15 µM, 2.8  ±  0.7 mM, respectively) [3]. Sigma-1 receptor agonists have been associated with neuroplasticity, neuroprotection, and cognitive function in the brain [21,22,23], and the sigma-2 receptor has been proposed as a potential therapeutic target for schizophrenia and Alzheimer’s disease [24]; however, their EEG effects are currently unclear. (R)-ketamine has also been proposed to induce greater serotonin (5-HT) release in the PFC compared to (S)-ketamine [25], although no difference was found between the two enantiomers’ binding affinity to 5-HT receptors [3], suggesting that higher 5-HT concentration may explain the stronger effect of (R)-ketamine. Activation of either 5-HT_2A_ or 5-HT_2B_ receptors has been shown to increase theta power [26,27].

This study focuses only on the EEG theta effects of a single shot of ketamine enantiomers. Future research might show further brain effects of ketamine enantiomers and molecular level experiments, possibly supported by behavioral effects of the drugs, using cognitive models.

## 4. Materials and Methods

### 4.1. Animals

Male Wistar rats (Han:WIST, Toxi-Coop, Budapest, Hungary) were kept under controlled environmental conditions at an ambient temperature of 21 °C ± 1 °C and a 12-h light/dark cycle (lights on at 10:00 AM). Standard rodent food and tap water were available ad libitum for the animals during the whole study. The sample size (6–8 rats/group) was determined based on previous research investigating the sleep effect of conventional antidepressants and ketamine enantiomers [6,14,15,28]. Before surgery, the rats were acclimatized to the housing conditions for 7 days and were handled for 2 min each day for 5 days during this period.

### 4.2. Surgery

Rats (9 weeks old), weighing between 290–325 g at surgery, were implanted with EEG and electromyographic (EMG) electrodes under 2% isoflurane anesthesia, using a Kopf stereotaxic instrument, as described earlier [6,27,28,29]. Briefly, for frontoparietal EEG recordings, stainless steel screw electrodes were placed epidurally over the left frontal cortex (1.5 mm lateral and 2.0 mm anterior to bregma) and over the left parietal cortex (1.5 mm lateral and 2.0 mm anterior to lambda), and a ground electrode was equipped over the cerebellum. Two EMG electrodes (spring electrodes made of stainless steel and embedded in silicon rubber; Plastics One Inc., Roanoke, VA, USA) were inserted into the neck’s musculature to record EMG signals.

Following a seven-day period of recovery, rats were relocated individually to a glass-covered square recording chamber. The rats remained in the chamber throughout the entire study, and they were connected to the EEG system through a flexible recording cable, which allowed them to move freely. Five days prior to the experiment, the animals were given intraperitoneal (i.p.) injections of physiological saline to acclimate them to the recording conditions. The quality and compliance of EEG and EMG signals were tested during the habituation period with signal tests.

### 4.3. Drugs and Treatments

The animals received 7.5, 15, or 30 mg/kg i.p. (R)-ketamine (Toronto Research Chemicals, Toronto, ON, Canada) or 7.5 or 15 mg/kg i.p. (S)-ketamine (Ketanest S, Pfizer Pharma GmbH, Berlin, Germany) or vehicle (saline) in a volume of 1 mL/kg body weight. All treatments were administered by a male researcher, while a female experimenter held the animals. In the EEG experiments, the drugs were given to each animal precisely at the start of the light (passive) phase. The timing of administration was chosen in accordance with earlier studies examining the impact of ketamine and conventional antidepressants on sleep [14,30,31]. The determination of the doses was based on our recent publication, in which we showed that the 15 mg/kg i.p. dose is relevant to show antidepressant effects of (S)-ketamine [6]. Therefore, in this study, we applied the same dose (15 mg/kg i.p.), a half dose (7.5 mg/kg i.p.), and a double dose (30 mg/kg i.p.) of (R)-ketamine and the same dose (15 mg/kg i.p.), and a half dose (7.5 mg/kg i.p.) of (S)-ketamine, to determine if the ketamine enantiomers have effects on EEG theta power.

### 4.4. EEG Recording and Analysis

After receiving treatment, EEG, EMG, and motor activity were monitored for 23 h (Coulburn Lablinc System, Holliston, MA, USA) while the animals were undisturbed. The signals were filtered below 0.50 Hz and above 100 Hz and were amplified (EMG: 5000 times, EEG: 10,000 times). Analog-to-digital conversion was carried out at a 256-Hz sampling rate.

Using standard criteria, the SleepSign for Animal R2.11.261.801 sleep analysis software (Kissei Comtec America Inc., Fort Lee, NJ, USA) was used to classify the sleep–wake stages [27]. First, we used the software’s automatic scoring feature to grade the vigilance stages. After that, researchers who were not aware of the rats’ treatment carried out visual supervision. The 4-s intervals, or epochs, were distinguished this way. The EEG activity during wakefulness was characterized by high EMG and motor activity, as well as low amplitude at the beta (14–29 Hz) and alpha (10–13 Hz) frequencies. The EEG showed minimal motor activity and reduced EMG activity during non-rapid eye movement (NREM) sleep, along with high-amplitude delta (0.5–4 Hz) frequency band activity and sporadic spindles (6–15 Hz). During REM sleep, EEG was characterized by low-amplitude, high-frequency activity and regular theta waves (5–9 Hz) without EMG activity or motor activity other than brief twitches.

For quantitative EEG (qEEG) theta power analysis, we used fast Fourier transformation (Hanning window, frequency resolution: 0.25 Hz) in the 5- to 9-Hz frequency range. The 1-Hz bins, marked by their upper limits, were created by summing the 0.25-Hz bins. Epochs with artifacts or stage transitions were excluded from the qEEG analysis.

### 4.5. Statistics

Theta EEG power was assessed during wakefulness and REM sleep but not during NREM sleep since this frequency band has been shown to be more important in these vigilance stages [14,15,16]. The statistical analysis was carried out and the graph bar figures were created using Prism 8 (GraphPad, San Diego, CA, USA). Power values for each frequency (5–9 Hz) were averaged per 6 h for each dose of (R)-ketamine and (S)-ketamine in wakefulness and REM sleep separately. Then, two-way ANOVA (two main factors: treatment and frequency) was performed on all three (R)-ketamine doses compared to saline and both (S)-ketamine doses compared to saline in wakefulness and REM sleep separately. For multiple comparisons, the Bonferroni post hoc test was carried out. A *p*-value of < 0.05 was accepted as statistically significant. Heat map figures were created using a custom script written in Matlab 9.13.0.2049777 (MathWorks, Natick, MA, USA) with the aim of visualizing data, but they do not demonstrate statistical results.

## 5. Conclusions

Our results show that (R)-ketamine but not (S)-ketamine has sustained effects on the EEG theta power in rats, which might contribute to and mark its unique hippocampal effects compared to (S)-ketamine in certain functions and animal models (e.g., cognitive impairment studies). Future studies should seek to explore the potential therapeutic effects and related mechanisms of action of (R)-ketamine.

## Data Availability

The datasets generated and analyzed during the current study are not publicly available due to ongoing analysis for future publication but are available from the corresponding author upon reasonable request.
